# Experimental Researches Applied to Physiology and Pathology

**Published:** 1853-01

**Authors:** E. Brown-Séquard

**Affiliations:** Paris


(To be continued.)

---

### *Experimental Researches applied to Physiology and Pathology.*

By E. Brown-Séquard, M. D., of Paris.

(Continued.)

### XXI.—On Muscular Irritability in Paralyzed Limbs, and its Semeiological Value.

Marshall Hall has published many papers, in which he has tried to prove that the degree of muscular irritability in paralyzed parts may be used as a means of diagnosis between *cerebral* and *spinal* paralysis.

He calls *cerebral* paralysis that in which the paralyzed part is deprived of the action of the brain, but not entirely, or not in the least, of the influence of the spinal cord. On the contrary, he calls *spinal* paralysis that in which the palsied part is altogether deprived of the action of both the brain and the spinal marrow. The cause of the cerebral paralysis may be seated either in the encephalon or the spinal cord; and the cause of the spinal paralysis may be seated either in the spinal cord or in the nerves.

In the same individual these two kinds of paralysis may exist together. Suppose a man in whom the brachial enlargement of the spinal cord is considerably softened, and consequently unable to act; the upper limbs then have a spinal paralysis, and the lower limbs, receiving their nerves from a healthy part of the spinal cord, have only a cerebral paralysis.

According to Marshall Hall, the cerebral paralysis is attended by augmented muscular irritability, and the spinal paralysis is attended by diminished irritability. He bases this opinion on the following experiments, and on some clinical observations.

On six frogs he divided the spinal marrow immediately below the origin of the brachial nerves; and he removed a portion of the ischiatic nerve of the right posterior extremity. He had immediately, or more remotely, the following interesting phenomena:

1st. The anterior extremities alone were moved spontaneously; both posterior extremities remaining entirely motionless when the animal, placed on its back, made ineffectual efforts to turn on the abdomen.

2d. Although perfectly paralytic in regard to spontaneous motion, the left posterior extremity, that still in connexion with the spinal marrow, moved very energetically when stimulated by pinching the toes with the forceps.

3d. The right posterior extremity, or that of which the ischiatic nerve was divided, was entirely paralytic, both in reference to spontaneous and excited motions.

4th. After the lapse of several weeks, whilst the muscular irritability of the left posterior extremity was gradually augmented, that of the right was gradually diminished,—phenomena observed when the animal was placed in water through which a slight galvanic shock was passed accurately in the direction of the mesial plane.

5th. Strychnine being now administered, the anterior extremities and the left posterior extremity, or that still in connexion with the spinal marrow, became affected with tetanus; but the right posterior extremity, or that severed from all nervous connexion with the spinal marrow, remained perfectly placid.

6th. Lastly, the difference in the degree of irritability in the muscular fibre of the two limbs was observed, when these were entirely separated from the rest of the animal.

After this exposition of the results of his experiments, Marshall Hall adds: "In a word, the muscles of the limb paralyzed by its separation from both cerebrum and spinal marrow, had lost their irritability; whilst those of the limb separated from its connexion with the cerebrum only, but left in connexion with the spinal marrow, not only retained their irritability, but probably possessed it in an augmented degree.*

It is easy to prove that Marshall Hall has been completely misled by his experiments.

It is well known that the more a muscle is excited, the more it contracts. As the degree of irritability is judged by the degree of the contraction, it follows that to know what is the degree of muscular irritability we ought to apply the same excitation to the muscles we desire to compare. In his experiments with galvanism and with strychnine, Marshall Hall has not done so. He has applied galvanism, so as to excite much more the muscles of the left side united with the spinal cord, than those of the right side.

The muscles of the left side were excited:

1st. Directly by the galvanic current.

2d. In consequence of the excitation of the motor nerves.

3d. In consequence of the excitation of the spinal cord, directly by the galvanic current, and secondarily in consequence of the excitation of the sensitive nerves.

So that the muscles on that side were moved not only by the direct excitation on them, but also by a reflex action, and in consequence of the direct excitation of the spinal cord.

As to the muscles of the right side, they were only excited by the small part of the galvanic current passing in them. During the

* On the diseases and derangements of the nervous system. 1841, p. 215.

first, and perhaps the second and the third week after the section of the ischiatic nerve, the muscles were also slightly excited by the motor fibres of that nerve, but after that time these fibres had lost their vital property, and were unable to excite a contraction in muscles.

From this analysis it results clearly that the mode of comparison of the two limbs, by the passage of a galvanic current, as it has been employed by Marshall Hall, could not decide in which side the muscles were more irritable.

The use of strychnine, also, could decide nothing in this question, because, as I have proved in a former article, this poison is not able to act upon muscles. It acts only on the nervous centres, and especially on the spinal cord. Therefore, the production of tetanus in one limb and not in the other, in the experiment of Marshall Hall, proves nothing at all as to the degree of muscular irritability.

To know what is that degree, it is necessary to separate the two limbs from the trunk, and then to excite directly the muscles. Marshall Hall has made this experiment, but he says nothing about the circumstances under which it was performed, and these circumstances were, as it will be shown, extremely important.

In my experiments, instead of dividing only the ischiatic nerve, I divided the four nerves going to one of the posterior limbs, of many frogs, in whom the spinal cord was divided immediately behind the roots of the brachial nerves.

I have found on the separated limbs of these frogs :

1st. That, at first, the muscular irritability was greater in the limb which had been deprived of the action of the spinal cord and of the brain, than in the limb deprived only of the action of the brain.

2d. That, at a variable time after the operation, the irritability was at the same degree in the two limbs.

3d. That, at last, the irritability became greater in the limb only deprived of the action of the brain, than in the other.*

* There is, in these experiments, a cause of error, arising from the existence on one side, and the absence, or, at least, a diminution in the other, of the vital power of the motor nerves ; but the difference is trifling when the nerves are divided very near their entrance in the muscles.

The differences in the degree of irritability have been observed: 1st. By the degree of the contraction under the influence of the same excitation; 2d. By the duration of irritability.

I have found that during a time, varying much according to seasons, and to many other circumstances, the muscular irritability increases in the two posterior limbs in a frog operated upon as I have described, and that the increase was more considerable in the limb where the nerves were divided than in the other.

If we compare two frogs, one operated on as before, and another having only had a division of all the nerves on one of the posterior limbs, we find, a few days after the operation, that in the four limbs separated from the body there are great differences as to the degree and the duration of muscular irritability: 1st. The three paralized limbs have a greater irritability than the one not at all paralyzed. From the three paralyzed limbs the two in which the nerves have been divided have both the same degree of irritability, and more than the limb in which there was only what Marshall Hall calls a cerebral paralysis. 2d. The irritability has lasted longer in the two limbs in which the nerves had been cut, than in the two other limbs; and from these two, that in which there was a cerebral paralysis has remained longer irritable.

If we examine the irritability in the posterior limbs of two frogs, operated on as aforesaid, for ten, twelve, or fifteen days, then we find that it is nearly at the same degree in the three paralyzed limbs, and greater there than in the non-paralyzed limb.

If the comparison is made four or five weeks after the operation, then the non-paralyzed limb has a greater irritability than the three paralyzed, and, from these three, the one deprived only of the cerebral action has a greater irritability than the two others.

The same experiments made on other animals than frogs, i. e. on guinea-pigs and rabbits, have given like results. I shall publish the details of these last experiments in a special paper, in which I intend to examine the value of the clinical observations of Marshall Hall, R. B. Todd, Duchenne de Boulogne, and others. I will merely state here, that in many cases it is almost impossible to know what is the difference in the degree of muscular irritability in a paralyzed limb, compared with a healthy limb, in a living man or animal. Galvanism and strychnine cannot give us any exact notion in this respect. I ought to add, that if we could know what is the relative degree of irritability in a paralyzed limb, we could not make use of that knowledge for the diagnosis of the seat of the alteration producing the paralysis. On the other side, we do not want to know what is the degree of irritability in order to establish such a diagnosis. It will be, almost constantly, easy to know whether a paralysis is a cerebral or a spinal one. The existence of reflex actions in the paralyzed parts, is sufficient to prove that there is a cerebral paralysis, and the absence or the slight degree of these actions will prove that there is a spinal paralysis.

The following conclusions may be drawn from the facts above related, and from others that I have not yet published.

1st. The degree of muscular irritability in paralyzed parts, becomes rapidly greater than in the healthy parts, but, after a variable length of time, it diminishes, and, as it is well known, it may disappear.

2d. The muscles deprived of the action of both the brain and the spinal marrow, become rapidly more irritable than the muscles deprived only of the action of the brain, but, after a certain time, there is also in them a more rapid diminution of irritability than in the others.

3d. It appears certain that the muscular irritability never disappears completely in parts deprived only of the cerebral action.*

4th. In certain cases of paralysis, and more particularly of the face, as after the removal of a large part of the facial nerve, the muscular irritability may exist for years, at least in rabbits and other animals.

5th. It is very difficult, and sometimes almost impossible, to know the relative degree of muscular irritability in healthy parts

*I have had a pigeon on which nearly an inch of the costal part of the spinal cord had been removed, and on which the muscular irritability in the posterior limbs, and a very great reflex power, have existed as long as I have taken care of it, *i. e.* more than twenty-seven months. I ought to say that there has been no re-union of the separated parts of the spinal cord.

compared with paralyzed parts, and such a knowledge could not be of a great semeiological value.

6th. The existence or the absence of reflex actions as a means of diagnosis between the cerebral and the spinal paralysis, has a much greater value than the degree of muscular irritability.

